# Mechanical memory in cells: mechanisms, effects, and applications in regenerative medicine

**DOI:** 10.3389/fbioe.2026.1922947

**Published:** 2026-08-12

**Authors:** Antara Bhuyan, Mark Ahearne

**Affiliations:** 1 Department of Mechanical, Manufacturing and Biomedical Engineering, School of Engineering, Trinity College Dublin, University of Dublin, Dublin, Ireland; 2 Trinity Centre for Biomedical Engineering, Trinity Biomedical Science Institute, Trinity College Dublin, University of Dublin, Dublin, Ireland

**Keywords:** biomaterials, mechanical memory, mechanobiology, mechanotransduction, stem cells, substrate stiffness

## Abstract

Mechanical memory is the ability of cells to retain information from past mechanical environments, which profoundly influences cellular behaviour and fate, particularly in stem cells. This review combines contemporary insights into mechanical memory with specific attention to its underlying processes, which span mechanotransduction to epigenetic alterations. Various approaches to study mechanical memory have been investigated through substrate stiffness modulation, shear stress, and cyclic strain while evaluating the impact of mechanical memory on several cell types. Applications in regenerative medicine are discussed, including stem cell expansion, tissue engineering, and disease treatment. Future research directions are proposed to enhance the understanding and control of mechanical memory for therapeutic advancements, addressing gaps in molecular mechanisms and clinical translation.

## 1 Introduction

Cells are known to alter their behaviour in response to differing mechanical environments. Numerous studies have shown that cell behaviour can be affected by changes in mechanical loading (Andreu et al., 2021; Hoffman et al., 2020), matrix stiffness (Discher et al., 2005; Masterton and Ahearne, 2019; Engler et al., 2006; Li T. et al., 2023; Jiang et al., 2024) and shear stress from fluid flow (Tzima et al., 2005; Dewey et al., 1981; Hu et al., 2024). In the body these changes can result from disease, aging, changes in lifestyle or physical activity. The physical cues experienced by the cells play an important role in shaping their function and fate. While many studies have examined the effect of these mechanical cues on changes in cells behaviour, fewer studies have focused on whether some or all these changes are transient or permanent once the mechanical cues are withdrawn or altered. The ability of cells to be permanently influenced by their mechanical environment is referred to as mechanical memory as the cells retain a memory of their previous mechanical environment even when the cells are removed from that environment.

Mechanical memory is important when trying to understand how cells respond to fluctuating mechanical cues in the body caused by physical activity, injury or disease and when trying to develop novel stem cell or tissue engineering based therapies to treat these conditions. Mechanical memory is particularly relevant in stem cell biology due to the sensitivity of stem cells to their mechanical environment. Substrate stiffness determines stem cell behavior by either preserving pluripotency or directing lineage-specific differentiation (Yang C. et al., 2014). However, stem cell behavior remains influenced by past exposure to stiff substrates through retention of mechanical memory, which can reduce their therapeutic usefulness even after the stimulus is removed (Gilbert et al., 2010). In regenerative medicine, traditional stiff culture dishes used to grow stem cells can cause them to develop scarring characteristics, which remain after transplantation and may lead to organ failure (Cao et al., 2022; Bouhrira et al., 2024). Thus, controlling mechanical memory is critical for maintaining stemness, directing differentiation, and enhancing the success of stem cell therapies.

## 2 Mechanisms of mechanical memory

**FIGURE 1:**
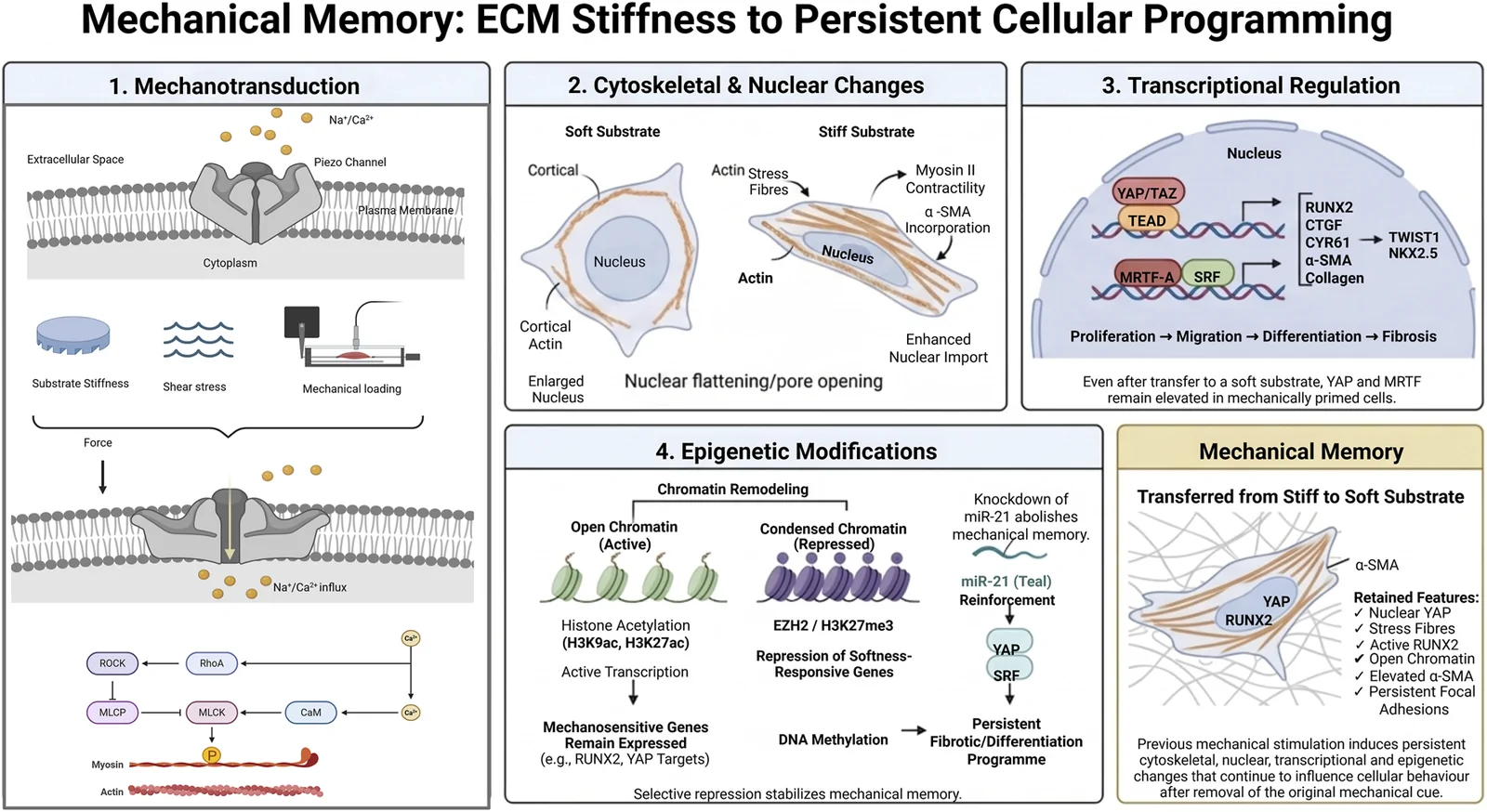
Mechanotransduction cascade leading to mechanical memory. Mechanical cues trigger opening of Piezo1 mechanosensitive channels, allowing Ca^2+^ influx and activation of calmodulin-dependent signaling and PKC pathways. These signals converge with integrin-mediated activation of RhoA/ROCK to enhance actin stress fiber formation and cytoskeletal tension. Forces transmitted to the nucleus via the LINC complex facilitate nuclear import of YAP/TAZ, which activate mechano-responsive transcription. Sustained activation induces chromatin modifications (e.g., increased H3K27ac, reduced repressive marks), resulting in stable transcriptional changes and the establishment of long-term mechanical memory (Created in BioRender. Ahearne, M. (2026) https://BioRender.com/y42i668).

Mechanical memory develops through intricate cellular processes that enable cells to detect mechanical stimuli and maintain responses over extended periods. These mechanisms play an essential role in the development of this phenomenon. An overview of these mechanisms is shown in Figure 1.

### 2.1 Mechanotransduction

The cellular detection of mechanical stimuli relies on integrin clusters, focal adhesions that convert matrix stiffness into biochemical signals to regulate downstream effectors, and the cytoskeleton (Chen et al., 2017). Examples include Rho/ROCK activation, Ca^2+^ influx through stretch-sensitive channels such as Piezo1. Increased extracellular matrix (ECM) rigidity promotes FAK and ILK signaling, RhoA-mediated stress fiber formation, and activation of MAPK pathways, ultimately leading to the nuclear translocation of YAP/TAZ and SRF/MRTF complexes (Chen et al., 2017). Persistent integrin signaling on a stiff matrix can thus “prime” cells by elevating nuclear YAP/TAZ and RUNX2 levels; indeed, human MSCs cultured on rigid tissue culture plastic (E∼3 GPa) for several days retain high YAP/TAZ and RUNX2 even after transfer to soft (∼2 kPa) hydrogels (Yang H. J. et al., 2014). Similarly, lung fibroblasts on stiff substrates maintain myofibroblastic markers (α-SMA, collagen) even when later placed on soft substrates (Balestrini et al., 2012). These examples illustrate how initial mechanotransduction events act as a memory of past stiffness exposure.

### 2.2 Cytoskeletal and nuclear changes

Mechanical forces cause the actin cytoskeleton to rearrange from a liquid state on soft substrates to a solid state on rigid substrates (Doss et al., 2020; Gupta et al., 2015). When fibroblasts grow on stiff substrates with a Young’s modulus of approximately 100 kPa they start to synthesize α-smooth muscle actin (αSMA) which serves to enhance cytoskeletal stability (Balestrini et al., 2012). Actomyosin contractility and stress fibers bear tension under stiff conditions; this tension is transmitted via the LINC complex to deform the nucleus and modulate nuclear pores (Berger et al., 2021). Persistent stress-fiber formation on stiff matrix increases nuclear lamina tension and pore opening, which enhances import of transcriptional regulators (Kureel et al., 2025). For instance, primed cells show enlarged nuclear area and elevated nesprin-2 expression, indicating sustained nuclear tension (Berger et al., 2021). Nuclear deformation also changes chromatin organization: heterochromatin domains can become condensed altering gene accessibility (Heo et al., 2015). Altogether, sustained actin tension and nuclear mechanics provide a physical “memory” scaffold that biases future gene expression, even after the stiffness cue is removed. The length of time that a cell is exposed to a particular stiffness is important in determining its behavior (Killaars et al., 2019).

### 2.3 Transcriptional regulation

Transcriptional regulation is a key memory effector. Mechanoresponsive transcription factors such as YAP/TAZ and serum response factor (SRF)–MRTF complexes shuttle in and out of the nucleus in response to substrate cues (Kureel et al., 2025). Under stiff conditions, YAP/TAZ translocate to the nucleus to activate pro-proliferative or lineage genes. Prolonged YAP nuclear localization leads to persistent transcriptional states, e.g., osteogenic RUNX2 expression in MSCs (Yang C. et al., 2014). Likewise, MRTF-A is released from G-actin under tension and enters the nucleus to co-activate SRF targets, including α-SMA and other contractility genes. Stiff-primed cells even after transfer to soft substrates retain elevated nuclear YAP/MRTF and enhanced focal adhesions, whereas knockdown of YAP erases this migratory “memory” in epithelial cells (Nasrollahi et al., 2017). Other transcription factors such as TWIST1, NKX2.5 and TGF-β family members also respond to mechanical cues; e.g., cyclic stretch drives nuclear localization of NKX2.5 (a cardiac transcription factor) and TWIST1, linking mechanical history to fate commitment (Kureel et al., 2025). Integrin-linked- kinase (ILK) has also been shown to play a role in YAP-mediated mechanical memory (He et al., 2023).

### 2.4 Epigenetic modifications

The maintenance of long-term mechanical memory depends on epigenetic changes such as histone methylation and acetylation that modify chromatin structures and regulate gene expression (Heo et al., 2015). The enzyme histone-lysine N-methyltransferase EZH2 facilitates rapid chromatin condensation in hMSCs by depositing repressive histone marks such as H3K27me3 on genes that would otherwise respond to softness when mechanical cues are present (Kureel et al., 2025; Heo et al., 2015). These molecular changes form enduring mechanical memory which affects cell fate determination. Studies show that stiff substrates with elasticity around 100 kPa cause elevated levels of histone acetylation markers H3K9ac and H3K27ac which leads to less chromatin condensation in hMSCs (Joshi et al., 2024). Human mesenchymal stem cells (hMSCs) grown on rigid tissue culture plates display increased histone acetylation with less compact chromatin compared to cells grown on softer materials which affects their stemness and aging processes (Joshi et al., 2024).

Additionally, stiff priming induces expression of mechanosensitive microRNAs (notably miR-21) that sustain profibrotic or differentiation programs (Kureel et al., 2025). In MSCs, miR-21 has been shown to be essential for maintaining mechanical memory of past stretch by reinforcing YAP/SRF signaling, thus its knockdown erases the memory of stiffness (Kureel et al., 2025). Persistent epigenetic remodeling via histone marks, DNA methylation or noncoding RNAs encodes the cell’s mechanical history, ensuring that gene-expression changes persist after the original cue is gone (Kureel et al., 2025).

## 3 Methods to study mechanical memory

Researchers have used different methods and materials designed to simulate physiological mechanical environments when studying mechanical memory. While some studies have developed mathematical models to predict cell behavior (Price et al., 2021), most studies involve directly evaluating cells isolated from tissues and their response to differing mechanical environments. The methods enable scientists to observe cellular responses and retention of mechanical memory. Some examples of these methods and what they have been used to discover are shown in Table 1.

**TABLE 1:** Methods and materials for studying mechanical memory.

Mechanical stimulus	Materials/Devices	Example studies and findings
Substrate stiffness modulation	• Polyacrylamide (E ≈ 0.1 kPa–100 kPa) (Tse and Engler, 2010) • PEG (E ≈ 1 kPa to 1 MPa) (Andreu et al., 2021; Lee et al., 2014) • PDMS (E ≈ 1 kPa to 4 MPa) (Masterton and Ahearne, 2019; Greaney et al., 2025)	• MSCs on stiff gels (≥10 kPa) maintain nuclear YAP/TAZ and osteogenic RUNX2 even after transfer to softer gel (Yang et al., 2014a) • Lung fibroblasts cultured on stiff surfaces differentiate into myofibroblasts (αSMA^+^) and retain this phenotype after softening (Balestrini et al., 2012) • Human ASCs primed on stiff plastic show ∼50% reduced adipogenic differentiation, which is not reversed by softening (Berger et al., 2021)
Fluidic shear stress	• Microfluidic parallel plate flow chamber • Perfusion systems	• Endothelial cells under sustained disturbed flow (atheroprone) exhibit long-lived pro-inflammatory gene expression, partly via sustained NF-κB activity (Nagel et al., 1999)
Cyclic Strain	• Stretchable silicone membranes (e.g., PDMS on Flexcell plates) • Custom built uniaxial or biaxial stretch bioreactors	• MSCs primed by cyclic stretch (10%, 1 Hz) show elevated sustained YAP nuclear localization and collagen expression (Kim et al., 2023b) • MSCs subjected cyclic stretch induces chromatin condensation via YAP activation (requiring Ca^2+^ signaling) (Heo et al., 2015)

### 3.1 Substrate stiffness modulation

The cell response to variations in substrate stiffness has been the most extensively examined approach to study mechanical memory. Synthetic hydrogels made from polymers including Polyethylene glycol (PEG) and polyacrylamide have been used to study mechanical memory due to their easily adjustable stiffness which ranges from soft at approximately 0.1–1 kPa to stiff at approximately 0.1–1 MPa (Tse and Engler, 2010; Lee et al., 2014). These biocompatible materials enable precise control of stiffness while often coated with ECM proteins to enable or enhance cell adhesion to the hydrogel surface. Cells are “primed” on hydrogels with a specific stiffness for hours or days, then transferred to a new stiffness to test for memory. In general, cells primed on high stiffness substrates show sustained cytoskeletal tension and transcriptional states (YAP+, α-SMA+) even when later transferred onto softer matrices (Yang C. et al., 2014; Kureel et al., 2025).

However, stiffness-based methods face several challenges. One challenge is the routine use of trypsin or other enzymes to detach, and re-seed cells can disrupt adhesion complexes and partially reset mechanotransduction, confounding the memory effect. In fact, standard passaging on rigid tissue-culture plastic itself imprints a “stiff memory” (Kureel et al., 2025). To overcome this limitation, new biomaterials that can alter their mechanical properties while cells remain attached have been developed. Magnetorheological elastomers have been produced that allow changes in material stiffness to occur via the application of a magnetic field (Bouhrira et al., 2025). The application of GelMA (Gelatin methacrylate) hydrogels have been explored as a method to study mechanical memory without the need to remove cells from the hydrogel surface, although these hydrogels can only increase in stiffness (Smits et al., 2024). Photoresponsive PEG and polyacrylamide base hydrogels have been developed to allow both increased and decreased in stiffness to occur when exposed to light at specific wavelengths (Madl et al., 2024; Ansari et al., 2025). Varying calcium ion concentrations can be used to modulate the stiffness of alginate hydrogels (Wei et al., 2020) although it may be difficult to decouple the effect on cells of changing stiffness and the increase in calcium.

Another limitation of many studies that examined the cells’ response to stiffness is that cells grow on planar 2D substrates that lack the native 3D tissue structure. *In vivo* cells usually reside within fibrous 3D matrices and contain cell-cell networks that modulate force transmission. Some hydrogels can be used to study the effect of stiffness on cells in a 3D matrix by embedding the cells within the hydrogel structure. Examples include collagen, agarose and alginate. However, the main limitation with these types of studies is that changes in hydrogel stiffness are often accompanied by changed in pore size, fiber network microarchitecture or matrix density. This makes it difficult to determine whether the changes in cell behavior are the result of the stiffness or the microstructure.

Another factor to consider when conducting stiffness studies is the duration and magnitude of “priming” and how this can influence memory. Cells cultured for excessively long periods or high-stiffness priming may induce senescence or irreversible fibrosis. For example. hMSCs cultured on initially stiff (E ≈ 10 kPa) and then softened (E ≈ 2 kPa) PEG hydrogels exhibited either reversible or irreversible YAP/TAZ activation, depending on the duration of high stiffness priming (Yang C. et al., 2014).

### 3.2 Fluidic shear stress

Fluid shear stress elicits long-lasting mechanobiological reprogramming in many cell types, especially endothelial cells since these cells are subjected to fluid flow in blood vessels (Tsaryk et al., 2022). In one study, human vascular endothelial cells exposed to laminar flow showed genome-wide chromatin remodelling with active enhancers (H3K27ac-marked) gained near flow‐upregulated genes and lost near downregulated genes and gained enhancers enriched for KLF-family transcription factor motifs while lost enhancers contain ETS motifs (Tsaryk et al., 2022). In line with this, Krüppel-like factors (KLF2/4) induced by shear recruit chromatin remodelers (e.g., SWI/SNF) to open enhancers of homeostatic genes such as BMPR2 and SMAD5 under laminar shear (Moonen et al., 2022). These studies show that even a transient flow exposure can “lock in” a protective gene program by epigenetic remodelling in endothelial cells.

Shear flow also reorganizes the cytoskeleton and nucleus in a flow dependent manner (Rojas-Gonzalez et al., 2023). Steady laminar flow induces endothelial cells elongation and alignment parallel to flow direction with robust stress fibers, whereas oscillatory or disturbed flow triggers a pro-inflammatory phenotype and prevents orderly alignment (Rojas-Gonzalez et al., 2023). Shear stress stimulates mechanosensitive ion channels (e.g., Piezo1, TRPV4) and downstream effectors: notably, oscillatory shear activates the Hippo pathway coactivators YAP/TAZ and relocalizes them to the nucleus, while laminar shear, via KLF2/4, induces anti-inflammatory and anti-oxidant transcriptional programs (Tsaryk et al., 2022; Rojas-Gonzalez et al., 2023).

Critically, some of these flow-induced changes are durable. Endothelial cell monolayers have been shown retain memory of prior flow when cells preconditioned with shear migrate faster and heal wounds more quickly than naive cells, implying a persistent wound-healing phenotype (Dessalles et al., 2021). Likewise, even sub-threshold shear downregulates inflammatory genes (VCAM1, SELE) and upregulates antioxidant defences at levels too low to induce cell elongation (Rojas-Gonzalez et al., 2023). These gene-expression shifts can outlast the shear stimulus, so that returned-to-static endothelial cells retain an anti-inflammatory, anti-thrombotic state (Rojas-Gonzalez et al., 2023; Dessalles et al., 2021). Together, these findings indicate that shear stress can condition cells via mechanosensitive pathways (YAP/TAZ, KLF, Ca^2+^ signalling) and chromatin modifiers (SWI/SNF, histone acetylation) to yield long-lived phenotypic changes (Tsaryk et al., 2022), with alignment and gene-expression adaptations relaxing only slowly after flow cessation.

### 3.3 Cyclic strain

To study cyclic strain and mechanical loading *in vitro*, commercial or custom-made bioreactors have been developed to mimic mechanically active tissues like muscle, tendon and bone (Carroll et al., 2017; Semodji et al., 2025; Riboh et al., 2008). In 2D cell culture or 3D tissues under uniaxial stretch, fibroblasts and other mesenchymal cells reorganize their cytoskeleton dramatically (Hoffman et al., 2020). Stress fibers reinforce and reorient, and new transmembrane actin–nuclear (TAN) lines form that tether F-actin to the nuclear envelope. Cyclic loading also triggers nuclear and epigenetic responses that encode memory. For instance, hMSCs subjected to cyclic tensile strain exhibited protein-level responses, including changes in the linker of nucleoskeleton and cytoskeleton (LINC complex, indicating mechanical memory (Gilbert et al., 2019). Rapid changes occur in the LINC complex due to high-intensity cyclic strain that specifically modifies Sad1/UNC-84 (SUN) domain-containing protein 2 (SUN2) to create reversible protein-level memory which affects nuclear mechanics and cell fate. Another study revealed that cyclic stretch impacts MSC alignment and smooth muscle differentiation and identified amplitude as a major factor affecting memory retention (Ghazanfari et al., 2009).

The persistence of stretch-induced effects depends on the duration and surrounding matrix. In 3D collagen tissues, short (∼1 h) static stretch had little long-term impacts, but 24 h of sustained stretching a permanent matrix softening and altered cell forces occurred (Hong et al., 2025). This means that only stretches above a certain threshold (in magnitude or duration) were imprinted.

## 4 Effects of mechanical memory on different cell types

A summary of how mechanical memory affects different cell types is shown in Table 2.

**TABLE 2:** Summary of key findings of mechanical memory effects on different cell types.

Cell type	Key effects of mechanical memory	References
Mesenchymal stem cells (MSCs) (BM-MSCs and ASCs)	• Stiff priming causes sustained nuclear YAP/TAZ and RUNX2 localization, biasing cells toward osteogenic lineage • Soft priming favors adipogenic differentiation • Long-term stiff culture increases nuclear Prelamin A and H3K27me3 marks, promoting senescence or reduced stemness • ASCs expanded on rigid plastic show impaired adipogenesis, enlarged tense nuclei, and upregulated osteogenic genes even after transfer to soft substrates • Stable memory requires ∼3–7 days of priming	Yang et al. (2014a), Berger et al. (2021), Kureel et al. (2025), Heo et al. (2015), Peng et al. (2017), Li et al. (2017), Park et al. (2011), Kureel et al. (2019), Heo et al. (2016), Schellenberg et al. (2011), Dunham et al. (2020)
Induced pluripotent stem cells (iPSCs)	• Mechanical priming (stiffness and cyclic strain) influences lineage commitment and retention (e.g., cardiomyocyte differentiation via NKX2.5 and YAP/TAZ) • Stiff/cardiac-like matrices or cyclic stretch enhance sarcomeric organization, contractile gene expression, and sustained beating after stimulus removal • Mechanical memory helps lock in lineage choices through chromatin remodeling	Kureel et al. (2025), Gwak et al., 2008; Chien (2008), Kim et al. (2023a), Lu et al. (2021)
Epithelial cells	• Stiff matrix priming results in faster migration, larger focal adhesions, increased stress fibers, and sustained nuclear YAP even on softer substrates • YAP is essential for this migratory memory • EMT programs triggered by rigid matrices can become irreversible due to epigenetic locking • Relevant in wound healing where prior stiff scar exposure maintains activation	Nasrollahi et al. (2017), Jain et al. (2023), Bhattacharya et al. (2023)
Cancer cells	• Stiff priming increases traction forces, collagen remodeling ability, and invasiveness that persists across softer environments • Sustained nuclear YAP and migratory phenotype promote metastasis • Glioblastoma cells retain migratory behavior from stiff ECM, driving invasion and recurrence • 2D culture imprints mechanical memory that alters cell behavior compared to 3D conditions	Cambria et al. (2024), Watson et al. (2021), Almeida et al. (2023), Chen et al. (2021), Suarez-Meade et al. (2025), Khan et al. (2026)

### 4.1 Mesenchymal stem cells

Bone marrow derived MSCs (BM-MSCs) are one of the highly characterized cell types when it comes to mechanical memory (Yang C. et al., 2014; Kureel et al., 2025; Heo et al., 2015; Peng et al., 2017; Li et al., 2017). These cells are highly mechano-sensitive and exhibit strong mechanical memory. Human BM-MSCs primed on very stiff plastic surfaces show prolonged nuclear localization of YAP/TAZ and RUNX2 even when moved to soft hydrogels (Yang C. et al., 2014). This “stiff-memory” biases them toward osteogenic lineage commitment. Conversely, BM-MSCs primed on soft matrices tend to favour adipogenic differentiation (Park et al., 2011; Kureel et al., 2019). Long-term culture on rigid substrates also increases nuclear prelamin A and H3K27me3 marks (Heo et al., 2016), locking in stemness or senescence programs (Schellenberg et al., 2011). Several studies have demonstrated that a minimal priming threshold (∼3–7 days on stiff matrix) is required for stable memory, after which cells resist reversion (Yang C. et al., 2014; Kureel et al., 2025). These findings suggest that BM-MSCs carry a record of past mechanical conditions, which influences their fate decisions.

Adipose-derived stem cells (ASCs) are a type of MSC that inhabit soft adipose tissue. *In vitro* expansion on standard plastic has been shown to impair their adipogenic function. Berger et al. (2021) found that ASCs serially passaged on rigid plastic reduced their ability to accumulate lipids, even if the final passage was performed on a soft substrate (Berger et al., 2021). Stiff-cultured ASCs developed larger, more tense nuclei (upregulating nesprin-2) and showed upregulation of osteogenic (RUNX2) genes at the expense of adipogenic ones. In another study, ASC primed on soft substrates delayed their differentiation to a pro-fibrotic phenotype (Dunham et al., 2020). These studies highlight that when cultivating ASCs substrate rigidity needs to be considered, particularly if the cells are to be used for clinical applications.

### 4.2 Induced pluripotent stem cells (iPSCs)

iPSCs differentiate into various lineages and thus are exposed to diverse mechanical environments depending on their fate. For example, cardiomyocyte-committed iPSCs experience cyclic strain and ∼10–20 kPa stiffness in the beating heart (Gwak et al., 2008), whereas iPSC-derived endothelial cells experience shear flow (Chien, 2008). Although direct “mechanical memory” studies in iPSCs are still emerging, evidence suggests mechanotransduction plays a key role in their fate retention (Gwak et al., 2008; Chien, 2008; Kim M. H. et al., 2023). Notably, mechanosensitive cardiac transcription factors such as NKX2.5 are regulated by matrix cues: cyclic stretch induces nuclear translocation of NKX2.5 in maturing cardiomyocytes, linking mechanical cues to gene expression (Kureel et al., 2025). One can speculate that iPSC-derived cardiomyocytes primed on a stiff cardiac-like matrix might retain higher NKX2.5/YAP activity and contractile gene expression even when later cultured on softer supports (Kureel et al., 2025). Indeed, prolonged stretch of cardiac progenitors has been shown to produce more robust sarcomere organization and sustained beating after stimulus removal (Lu et al., 2021). In general, mechanical priming of pluripotent cells tends to lock in lineage choices: e.g., stiffer matrices drive more cardiomyogenic and osteogenic differentiation, a memory of which persists on softer gels (likely via YAP/TAZ-mediated chromatin remodelling) (Yang C. et al., 2014; Kureel et al., 2025). Further studies are needed, but current data imply iPSC fate can be “mechanically programmed” through stiffness and stretch priming.

### 4.3 Epithelial cells

Recent work shows that epithelial sheets have mechanical memory in their migratory behaviour (Nasrollahi et al., 2017). For instance, Nasrollahi et al. (2017) demonstrated that epithelial cells primed on a stiff matrix migrate significantly faster and maintain nuclear YAP after moving onto a softer matrix, compared to cells always on a soft matrix (Nasrollahi et al., 2017). The primed cells formed larger focal adhesions and more stress fibers. Importantly, silencing YAP abrogated this effect, indicating YAP is essential for epithelial mechanical memory. In physiological contexts such as wound healing, this suggests that epithelial cells previously exposed to stiff scar tissue might remain activated after ECM remodelling (Nasrollahi et al., 2017; Jain et al., 2023). Similarly, epithelial–mesenchymal transition (EMT) programs, once initiated by rigid matrices, can be irreversible due to epigenetic locking of gene expression (Bhattacharya et al., 2023).

### 4.4 Cancer cells

Emerging evidence indicates that cancer cells acquire a mechanical memory that influences invasiveness and colonization (Cambria et al., 2024; Watson et al., 2021). Mechanically primed cells, such as those subjected to a stiff matrix, generate higher traction forces and more effectively remodel 3D collagen fibers during invasion (Almeida et al., 2023). Almeida et al. (2023) showed that fibroblasts previously cultured on stiff matrices exert greater force to align collagen and invade softer microenvironments than unprimed cells (Almeida et al., 2023). Likewise, breast carcinoma cells grown on rigid collagen show sustained nuclear YAP and migratory behaviour upon transfer to soft matrix, promoting metastasis potential (Chen et al., 2021). Glioblastoma cells have demonstrated mechanical memory in modulating cell behaviour and supporting tumour growth. Increases in ECM stiffness promote a migratory cell phenotype that is maintained upon re-exposure to a softer matrix (Suarez-Meade et al., 2025). Since the stiffness of glioblastoma ECM increases the further it is from the tumor core, this promotes cells to migration from the core to the tumor surface where they can continue to proliferate and migrate to other sites (Suarez-Meade et al., 2025). Mechanical memory has also impeded attempts to generate *in-vitro* models of glioblastoma as and when the cells are cultured on a 2D substrate, they alter their behaviour and retain memory of this environment (Khan et al., 2026).

## 5 Applications of mechanical memory in regenerative medicine

Mechanical preconditioning of stem cells on appropriately tuned substrates can preserve their ‘stemness’ during long-term culture. For example, neural-crest-derived dental pulp stem cells (DPSC) lose potency during expansion on flat plastic, but training them on high‐aspect‐ratio micropatterns (“stem cell gym”) dramatically enhances the expression of pluripotency markers (Sox2, Nanog) and differentiation potential (Li C. et al., 2023). This micropattern training remodels the cytoskeleton and nucleus, activates pro‐stemness kinases (ERK/STAT3) and inhibits YAP nuclear translocation, which together lock in a stem‐like transcriptional program. Similarly, soft 2D substrates or dynamic stiffness can prevent loss of self-renewal; stem cells expanded on compliant matrices maintain higher proliferation and multilineage potential than cells on rigid plastic (Kureel et al., 2025). Overall, mechanical training can maintain stem‐cell identity without genetic manipulation, yielding large numbers of viable cells for therapy (Kureel et al., 2025; Li C. et al., 2023).

Mechanical memory can reduce the need for additional growth factors. In the DPSC “stem cell gym” system, enhanced stemness and regenerative function were achieved without adding any growth factors to the culture medium (Li C. et al., 2023). Likewise, synovial MSCs preconditioned by cyclic tensile strain showed meniscal repair capacity equivalent to that of cells treated with pro-chondrogenic growth factors (Ai et al., 2025). In effect, mechanical “priming” acts like a surrogate niche signal: it induces autocrine and intracellular responses that bias cells toward proliferation and differentiation (Li C. et al., 2023; Ai et al., 2025). By tuning substrate stiffness or applying controlled strain, cells can be pre-activated so that fewer bioactive molecules are needed post-transplant. This has practical advantages for cell manufacturing since it can lower cost and simplify protocols. Importantly, mechanically primed MSCs still secrete beneficial paracrine factors, but the mechanical stimulus enables them to function optimally even in the absence of added cytokines (Li C. et al., 2023; Ai et al., 2025).

In tissue engineering, next-generation biomaterial scaffolds that utilize mechanical memory are being engineered to optimize cell behaviour and tissue formation. One example is dynamic hydrogels whose stiffness can be changed on demand. The ability to control mechanical memory through magnetic nanofibrous hydrogels that stiffen in reaction to magnetic fields results in improved MSC osteogenesis while reducing adipogenesis (Islam et al., 2023). Other groups have developed “click‐bond” hydrogels that covalently link to cell integrins, ensuring persistent force transmission *in vivo*. In a recent study, such integrin‐binding hydrogels elicited a robust regenerative response from transplanted skeletal muscle cells by transmitting endogenous stress through the linkage, demonstrating that scaffold design can directly activate mechanosensitive pathways *in vivo* (Ueda et al., 2025).

## 6 Conclusion and future directions

Mechanical memory within cells represents a dynamic process that affects stem cell differentiation, tissue engineering practices, and how diseases progress. Mechanically “trained” cells tend to engraft more reliably, produce less scarring, and can be expanded without high doses of costly growth factors. Mechanical reprogramming is non-genetic and adjustable on-the-fly (by changing matrix stiffness or stretch), making it safer and tuneable. Furthermore, it taps into the cell’s own regulatory networks (YAP/TAZ, MRTF-A, epigenetic machinery) to achieve lasting reprogramming. As the field matures, mechanical memory may prove as important as growth factors or genetic factors in defining cell fate, offering an orthogonal strategy to shape cells for therapy.

## References

[B1] Ai L. Shi Q. Du M. Wang Y. Zhang Z. Du J. (2025). Stem cell warm-up via biomimetic mechanical priming enhances synovial-derived MSCs adaptation for meniscal repair. Osteoarthr. Cartil. 33 (10), 1200–1212. 10.1016/j.joca.2025.07.001 40623448

[B2] Almeida J. A. Mathur J. Lee Y. L. Sarker B. Pathak A. (2023). Mechanically primed cells transfer memory to fibrous matrices for invasion across environments of distinct stiffness and dimensionality. Mol. Biol. Cell 34 (6), ar54. 10.1091/mbc.e22-10-0469 36696158 PMC10208097

[B3] Andreu I. Falcones B. Hurst S. Chahare N. Quiroga X. Le Roux A. L. (2021). The force loading rate drives cell mechanosensing through both reinforcement and cytoskeletal softening. Nat. Commun. 12 (1), 4229. 10.1038/s41467-021-24383-3 34244477 PMC8270983

[B4] Ansari A. Bhowmik S. Zhang K. Reeves C. L. Vahala D. Choi Y. S. (2025). A visible light-responsive hydrogel to study the effect of dynamic tissue stiffness on cellular mechanosensing. Adv. Funct. Mater. 35 (35), 2501585. 10.1002/adfm.202501585

[B5] Balestrini J. L. Chaudhry S. Sarrazy V. Koehler A. Hinz B. (2012). The mechanical memory of lung myofibroblasts. Integr. Biol. (Camb) 4 (4), 410–421. 10.1039/c2ib00149g 22410748

[B6] Berger A. J. Anvari G. Bellas E. (2021). Mechanical memory impairs Adipose-derived stem cell (ASC) adipogenic capacity after long-term *in vitro* expansion. Cell Mol. Bioeng. 14 (5), 397–408. 10.1007/s12195-021-00705-9 34777600 PMC8548437

[B7] Bhattacharya S. Mukherjee A. Pisano S. Dimri S. Knaane E. Altshuler A. (2023). The biophysical property of the limbal niche maintains stemness through YAP. Cell Death Differ. 30 (6), 1601–1614. 10.1038/s41418-023-01156-7 37095157 PMC10244376

[B8] Bouhrira N. Vite A. Margulies K. B. (2024). Distinct cytoskeletal regulators of mechanical memory in cardiac fibroblasts and cardiomyocytes. Basic Res. Cardiol. 119 (2), 277–289. 10.1007/s00395-023-01030-0 38349539 PMC13725860

[B9] Bouhrira N. Pizarro S. Senf V. Margulies K. B. (2025). Matrix stiffening induces mechanical memory and nuclear fragility in cardiomyocytes via microtubule-lamin coupling. bioRxiv, 2025: 07.17.665449. 10.1101/2025.07.17.665449 40777352 PMC12330650

[B10] Cambria E. Coughlin M. F. Floryan M. A. Offeddu G. S. Shelton S. E. Kamm R. D. (2024). Linking cell mechanical memory and cancer metastasis. Nat. Rev. Cancer 24 (3), 216–228. 10.1038/s41568-023-00656-5 38238471 PMC11146605

[B11] Cao H. Zhou Q. Liu C. Zhang Y. Xie M. Qiao W. (2022). Substrate stiffness regulates differentiation of induced pluripotent stem cells into heart valve endothelial cells. Acta Biomater. 143, 115–126. 10.1016/j.actbio.2022.02.032 35235867

[B12] Carroll S. F. Buckley C. T. Kelly D. J. (2017). Cyclic tensile strain can play a role in directing both intramembranous and endochondral ossification of mesenchymal stem cells. Front. Bioeng. Biotechnol. 5, 73. 10.3389/fbioe.2017.00073 29230389 PMC5712005

[B13] Chen Y. Ju L. Rushdi M. Ge C. Zhu C. (2017). Receptor-mediated cell mechanosensing. Mol. Biol. Cell 28 (23), 3134–3155. 10.1091/mbc.E17-04-0228 28954860 PMC5687017

[B14] Chen W. Park S. Patel C. Bai Y. Henary K. Raha A. (2021). The migration of metastatic breast cancer cells is regulated by matrix stiffness via YAP signalling. Heliyon 7 (2), e06252. 10.1016/j.heliyon.2021.e06252 33659755 PMC7895759

[B15] Chien S. (2008). Role of shear stress direction in endothelial mechanotransduction. Mol. Cell Biomech. 5 (1), 1–8. 10.3970/mcb.2008.005.001 18524241

[B16] Dessalles C. A. Leclech C. Castagnino A. Barakat A. I. (2021). Integration of substrate- and flow-derived stresses in endothelial cell mechanobiology. Commun. Biol. 4 (1), 764. 10.1038/s42003-021-02285-w 34155305 PMC8217569

[B17] Dewey C. F. Jr. Bussolari S. R. Gimbrone M. A. Davies P. F. (1981). The dynamic response of vascular endothelial cells to fluid shear stress. J. Biomech. Eng. 103 (3), 177–185. 10.1115/1.3138276 7278196

[B18] Discher D. E. Janmey P. Wang Y. L. (2005). Tissue cells feel and respond to the stiffness of their substrate. Science 310 (5751), 1139–1143. 10.1126/science.1116995 16293750

[B19] Doss B. L. Pan M. Gupta M. Grenci G. Mège R. M. Lim C. T. (2020). Cell response to substrate rigidity is regulated by active and passive cytoskeletal stress. Proc. Natl. Acad. Sci. U. S. A. 117 (23), 12817–12825. 10.1073/pnas.1917555117 32444491 PMC7293595

[B20] Dunham C. Havlioglu N. Chamberlain A. Lake S. Meyer G. (2020). Adipose stem cells exhibit mechanical memory and reduce fibrotic contracture in a rat elbow injury model. FASEB J. 34 (9), 12976–12990. 10.1096/fj.202001274R 33411380 PMC8745456

[B21] Engler A. J. Sen S. Sweeney H. L. Discher D. E. (2006). Matrix elasticity directs stem cell lineage specification. Cell 126 (4), 677–689. 10.1016/j.cell.2006.06.044 16923388

[B22] Ghazanfari S. Tafazzoli-Shadpour M. Shokrgozar M. A. (2009). Effects of cyclic stretch on proliferation of mesenchymal stem cells and their differentiation to smooth muscle cells. Biochem. Biophys. Res. Commun. 388 (3), 601–605. 10.1016/j.bbrc.2009.08.072 19695226

[B23] Gilbert P. M. Havenstrite K. L. Magnusson K. E. G. Sacco A. Leonardi N. A. Kraft P. (2010). Substrate elasticity regulates skeletal muscle stem cell self-renewal in culture. Science 329 (5995), 1078–1081. 10.1126/science.1191035 20647425 PMC2929271

[B24] Gilbert H. T. J. Mallikarjun V. Dobre O. Jackson M. R. Pedley R. Gilmore A. P. (2019). Nuclear decoupling is part of a rapid protein-level cellular response to high-intensity mechanical loading. Nat. Commun. 10 (1), 4149. 10.1038/s41467-019-11923-1 31515493 PMC6742657

[B25] Greaney A. J. McCarthy C. M. Vethil J. P. Abubaker M. Reardon E. C. Crowley F. D. (2025). A comprehensive protocol for PDMS fabrication for use in cell culture. PLoS One 20 (5), e0323283. 10.1371/journal.pone.0323283 40354467 PMC12068733

[B26] Gupta M. Sarangi B. R. Deschamps J. Nematbakhsh Y. Callan-Jones A. Margadant F. (2015). Adaptive rheology and ordering of cell cytoskeleton govern matrix rigidity sensing. Nat. Commun. 6, 7525. 10.1038/ncomms8525 26109233 PMC4599139

[B27] Gwak S. J. Bhang S. H. Kim I. K. Kim S. S. Cho S. W. Jeon O. (2008). The effect of cyclic strain on embryonic stem cell-derived cardiomyocytes. Biomaterials 29 (7), 844–856. 10.1016/j.biomaterials.2007.10.050 18022225

[B28] He C. Wang T. Wang Y. Xu T. Zhao S. Shi H. (2023). ILK regulates osteogenic differentiation of human periodontal Ligament stem cells through YAP-mediated mechanical memory. Oral Dis. 29 (1), 274–284. 10.1111/odi.13997 34370371

[B29] Heo S. J. Thorpe S. D. Driscoll T. P. Duncan R. L. Lee D. A. Mauck R. L. (2015). Biophysical regulation of chromatin architecture instills a mechanical memory in mesenchymal stem cells. Sci. Rep. 5, 16895. 10.1038/srep16895 26592929 PMC4655352

[B30] Heo S. J. Driscoll T. P. Thorpe S. D. Nerurkar N. L. Baker B. M. Yang M. T. (2016). Differentiation alters stem cell nuclear architecture, mechanics, and mechano-sensitivity. Elife 5, e18207. 10.7554/eLife.18207 27901466 PMC5148611

[B31] Hoffman L. M. Smith M. A. Jensen C. C. Yoshigi M. Blankman E. Ullman K. S. (2020). Mechanical stress triggers nuclear remodeling and the formation of transmembrane actin nuclear lines with associated nuclear pore complexes. Mol. Biol. Cell 31 (16), 1774–1787. 10.1091/mbc.E19-01-0027 31967947 PMC7521858

[B32] Hong Y. Peng X. Yu H. Jafari M. Shakiba D. Huang Y. (2025). Cell-matrix feedback controls stretch-induced cellular memory and fibroblast activation. Proc. Natl. Acad. Sci. U. S. A. 122 (12), e2322762122. 10.1073/pnas.2322762122 40100625 PMC11962495

[B33] Hu S. Y. Xue C. D. Li Y. J. Li S. Gao Z. N. Qin K. R. (2024). Microfluidic investigation for shear-stress-mediated repair of dysglycemia-induced endothelial cell damage. Mechanobiol. Med. 2 (3), 100069. 10.1016/j.mbm.2024.100069 40395495 PMC12082321

[B34] Islam M. S. Molley T. G. Hung T. t. Sathish C. I. Putra V. D. L. Jalandhra G. K. (2023). Magnetic nanofibrous hydrogels for dynamic control of stem cell differentiation. ACS Appl. Mater. Interfaces 15 (44), 50663–50678. 10.1021/acsami.3c07021 37643902

[B35] Jain P. Corbo S. Mohammad K. Sahoo S. Ranganathan S. George J. T. (2023). Epigenetic memory acquired during long-term EMT induction governs the recovery to the epithelial state. J. R. Soc. Interface 20 (198), 20220627. 10.1098/rsif.2022.0627 36628532 PMC9832289

[B36] Jiang T. Zheng M. T. Li R. M. Ouyang N. J. (2024). The effects of matrix stiffness on immune cells in bone biology. Mechanobiol. Med. 2 (2), 100046. 10.1016/j.mbm.2024.100046 40395853 PMC12082311

[B37] Joshi R. Suryawanshi T. Mukherjee S. Shukla S. Majumder A. (2024). Chromatin condensation delays senescence in human mesenchymal stem cells by safeguarding nuclear damages during *in vitro* expansion. J. Tissue Eng. Regen. Med. 2024, 1543849. 10.1155/2024/1543849 40225747 PMC11919206

[B38] Khan M. Kollenz P. Fritzenschaft M. Taheri F. Colombo F. Blumberg J. W. (2026). Dimensional memory in glioblastoma mechanics: traction force analysis of cells cultured in 2D versus 3D collagen environments. Bioact. Mater 55, 515–528. 10.1016/j.bioactmat.2025.09.025 41078868 PMC12514354

[B39] Killaars A. R. Grim J. C. Walker C. J. Hushka E. A. Brown T. E. Anseth K. S. (2019). Extended exposure to stiff microenvironments leads to persistent chromatin remodeling in human mesenchymal stem cells. Adv. Sci. (Weinh) 6 (3), 1801483. 10.1002/advs.201801483 30775233 PMC6364489

[B40] Kim M. H. Thanuthanakhun N. Kino-Oka M. (2023a). Novel strategy to improve hepatocyte differentiation stability through synchronized behavior-driven mechanical memory of iPSCs. Biotechnol. Bioeng. 120 (2), 593–607. 10.1002/bit.28285 36369977

[B41] Kim E. Riehl B. D. Bouzid T. Yang R. Duan B. Donahue H. J. (2023b). YAP mechanotransduction under cyclic mechanical stretch loading for mesenchymal stem cell osteogenesis is regulated by ROCK. Front. Bioeng. Biotechnol. 11, 1306002. 10.3389/fbioe.2023.1306002 38274006 PMC10809151

[B42] Kureel S. K. Mogha P. Khadpekar A. Kumar V. Joshi R. Das S. (2019). Soft substrate maintains proliferative and adipogenic differentiation potential of human mesenchymal stem cells on long-term expansion by delaying senescence. Biol. Open 8 (4), bio039453. 10.1242/bio.039453 31023646 PMC6503999

[B43] Kureel S. K. Maroto R. Davis K. Sheetz M. (2025). Cellular mechanical memory: a potential tool for mesenchymal stem cell-based therapy. Stem Cell Res. Ther. 16 (1), 159. 10.1186/s13287-025-04249-x 40165288 PMC11960036

[B44] Lee S. Tong X. Yang F. (2014). The effects of varying poly(ethylene glycol) hydrogel crosslinking density and the crosslinking mechanism on protein accumulation in three-dimensional hydrogels. Acta Biomater. 10 (10), 4167–4174. 10.1016/j.actbio.2014.05.023 24887284

[B45] Li C. X. Talele N. P. Boo S. Koehler A. Knee-Walden E. Balestrini J. L. (2017). MicroRNA-21 preserves the fibrotic mechanical memory of mesenchymal stem cells. Nat. Mater. 16 (3), 379–389. 10.1038/nmat4780 27798620

[B46] Li T. Li Y. Wu H. Peng C. Wang J. Chen S. (2023a). Extracellular cell matrix stiffness-driven drug resistance of breast cancer cells via EGFR activation. Mechanobiol. Med. 1 (2), 100023. 10.1016/j.mbm.2023.100023 40395635 PMC12082153

[B47] Li C. Meng F. Yang Z. Peng J. Guo Y. Na J. (2023b). Micropattern-based stem cell gym: mechanical memory enhanced stemness maintenance of human dental pulp stem cells and nerve regeneration. Adv. Funct. Mater. 33 (49), 2302829. 10.1002/adfm.202302829

[B48] Lu K. Seidel T. Cao-Ehlker X. Dorn T. Batcha A. M. N. Schneider C. M. (2021). Progressive stretch enhances growth and maturation of 3D stem-cell-derived myocardium. Theranostics 11 (13), 6138–6153. 10.7150/thno.54999 33995650 PMC8120210

[B49] Madl C. M. Wang Y. X. Holbrook C. A. Su S. Shi X. Byfield F. J. (2024). Hydrogel biomaterials that stiffen and soften on demand reveal that skeletal muscle stem cells harbor a mechanical memory. Proc. Natl. Acad. Sci. U. S. A. 121 (35), e2406787121. 10.1073/pnas.2406787121 39163337 PMC11363279

[B50] Masterton S. Ahearne M. (2019). Influence of polydimethylsiloxane substrate stiffness on corneal epithelial cells. R. Soc. Open Sci. 6, 191796. 10.1098/rsos.191796 31903218 PMC6936283

[B51] Moonen J. R. Chappell J. Shi M. Shinohara T. Li D. Mumbach M. R. (2022). KLF4 recruits SWI/SNF to increase chromatin accessibility and reprogram the endothelial enhancer landscape under laminar shear stress. Nat. Commun. 13 (1), 4941. 10.1038/s41467-022-32566-9 35999210 PMC9399231

[B52] Nagel T. Resnick N. Dewey C. F. Gimbrone M. A. (1999). Vascular endothelial cells respond to spatial gradients in fluid shear stress by enhanced activation of transcription factors. Arterioscler. Thromb. Vasc. Biol. 19 (8), 1825–1834. 10.1161/01.atv.19.8.1825 10446060

[B53] Nasrollahi S. Walter C. Loza A. J. Schimizzi G. V. Longmore G. D. Pathak A. (2017). Past matrix stiffness primes epithelial cells and regulates their future collective migration through a mechanical memory. Biomaterials 146, 146–155. 10.1016/j.biomaterials.2017.09.012 28918264 PMC5659718

[B54] Park J. S. Chu J. S. Tsou A. D. Diop R. Tang Z. Wang A. (2011). The effect of matrix stiffness on the differentiation of mesenchymal stem cells in response to TGF-beta. Biomaterials 32 (16), 3921–3930. 10.1016/j.biomaterials.2011.02.019 21397942 PMC3073995

[B55] Peng T. Liu L. MacLean A. L. Wong C. W. Zhao W. Nie Q. (2017). A mathematical model of mechanotransduction reveals how mechanical memory regulates mesenchymal stem cell fate decisions. BMC Syst. Biol. 11 (1), 55. 10.1186/s12918-017-0429-x 28511648 PMC5434622

[B56] Price C. C. Mathur J. Boerckel J. D. Pathak A. Shenoy V. B. (2021). Dynamic self-reinforcement of gene expression determines acquisition of cellular mechanical memory. Biophys. J. 120 (22), 5074–5089. 10.1016/j.bpj.2021.10.006 34627766 PMC8633715

[B57] Riboh J. Chong A. K. S. Pham H. Longaker M. Jacobs C. Chang J. (2008). Optimization of flexor tendon tissue engineering with a cyclic strain bioreactor. J. Hand Surg. Am. 33 (8), 1388–1396. 10.1016/j.jhsa.2008.04.019 18929207

[B58] Rojas-Gonzalez D. M. Babendreyer A. Ludwig A. Mela P. (2023). Analysis of flow-induced transcriptional response and cell alignment of different sources of endothelial cells used in vascular tissue engineering. Sci. Rep. 13 (1), 14384. 10.1038/s41598-023-41247-6 37658092 PMC10474151

[B59] Schellenberg A. Lin Q. Schüler H. Koch C. M. Joussen S. Denecke B. (2011). Replicative senescence of mesenchymal stem cells causes DNA-methylation changes which correlate with repressive histone marks. Aging (Albany NY) 3 (9), 873–888. 10.18632/aging.100391 22025769 PMC3227452

[B60] Semodji A. M. Everingham J. B. Hollar K. A. Siegel D. N. Jamison S. E. Wilder F. R. (2025). A multiaxial bioreactor system that applies targeted magnitudes of strain energy to 3D cellular constructs. J. Mech. Behav. Biomed. Mater. 168, 106983. 10.1016/j.jmbbm.2025.106983 40222321

[B61] Smits J. van der Pol A. Goumans M. J. Bouten C. V. C. Jorba I. (2024). GelMA hydrogel dual photo-crosslinking to dynamically modulate ECM stiffness. Front. Bioeng. Biotechnol. 12, 1363525. 10.3389/fbioe.2024.1363525 38966190 PMC11222782

[B62] Suarez-Meade P. Whitehead R. Rosenfeld S. Schiapparelli P. Konstantopoulos K. Quinones-Hinojosa A. (2025). Extracellular matrix stiffness conditions glioblastoma cells for long-term migration: mechanical memory as a driver of invasion and recurrence in glioblastoma. Neuro Oncol. 28 (1), 19–37. 10.1093/neuonc/noaf205 40973065 PMC12962637

[B63] Tsaryk R. Yucel N. Leonard E. V. Diaz N. Bondareva O. Odenthal-Schnittler M. (2022). Shear stress switches the association of endothelial enhancers from ETV/ETS to KLF transcription factor binding sites. Sci. Rep. 12 (1), 4795. 10.1038/s41598-022-08645-8 35314737 PMC8938417

[B64] Tse J. R. Engler A. J. (2010). Preparation of hydrogel substrates with tunable mechanical properties. Curr. Protoc. Cell Biol. 47, 10.16.1–10.16.16. 10.1002/0471143030.cb1016s47 20521229

[B65] Tzima E. Irani-Tehrani M. Kiosses W. B. Dejana E. Schultz D. A. Engelhardt B. (2005). A mechanosensory complex that mediates the endothelial cell response to fluid shear stress. Nature 437 (7057), 426–431. 10.1038/nature03952 16163360

[B66] Ueda N. Okazaki H. Mikuma A. Kunieda A. Kawashima S. Torii T. (2025). *In vivo* mechano-tissue engineering by hydrogels capable of transmitting intercellular mechanical stress. Nat. Commun. 16 (1), 8847. 10.1038/s41467-025-64656-9 41093855 PMC12528752

[B67] Watson A. W. Grant A. D. Parker S. S. Hill S. Whalen M. B. Chakrabarti J. (2021). Breast tumor stiffness instructs bone metastasis via maintenance of mechanical conditioning. Cell Rep. 35 (13), 109293. 10.1016/j.celrep.2021.109293 34192535 PMC8312405

[B68] Wei D. Liu A. Sun J. Chen S. Wu C. Zhu H. (2020). Mechanics-controlled dynamic cell niches guided osteogenic differentiation of stem cells via preserved cellular mechanical memory. ACS Appl. Mater Interfaces 12 (1), 260–274. 10.1021/acsami.9b18425 31800206

[B69] Yang C. Tibbitt M. W. Basta L. Anseth K. S. (2014a). Mechanical memory and dosing influence stem cell fate. Nat. Mater 13 (6), 645–652. 10.1038/nmat3889 24633344 PMC4031270

[B70] Yang H. J. Kim K. J. Kim M. K. Lee S. J. Ryu Y. H. Seo B. F. (2014b). The stem cell potential and multipotency of human adipose tissue-derived stem cells vary by cell donor and are different from those of other types of stem cells. Cells Tissues Organs 199 (5-6), 373–383. 10.1159/000369969 25823468

